# Effects of Physical Exercise Combined with Accommodative Training on Visual Acuity and Ocular Biometric Parameters in Children Aged 6–7

**DOI:** 10.3390/life16091529

**Published:** 2026-09-15

**Authors:** Miyu Wang, Rongbin Yin, Haijie Shi, Guiming Zhu, Kaixin Niu, Jue Wang, Beili Wu, Caimei Bai

**Affiliations:** 1 Physical Education and Sports School, Soochow University, Suzhou 215021, China; micoraline@163.com (M.W.); yrb@suda.edu.cn (R.Y.); shihaijie2022@163.com (H.S.); zhuguiming918@163.com (G.Z.);; 2 College of Physical Education, Shaanxi Xueqian Normal University, Xi’an 710100, China

**Keywords:** children, physical exercise, accommodative training, visual acuity, ocular biometric parameters

## Abstract

Promoting visual health in early childhood is crucial for visual development, and effective school-based interventions are urgently needed. This study examined the effects of physical exercise combined with accommodative training on visual acuity and ocular biometric parameters in children aged 6–7 years. A total of 163 first-grade students were allocated by intact classes to an experimental group (*n* = 82), which received a 32-week physical exercise program incorporating visual tasks designed to stimulate ciliary-muscle accommodation, or a control group (n = 81), which received standard physical education. Uncorrected distance visual acuity (UDVA), kinetic visual acuity (KVA), axial length (AL), corneal curvature (CC), and anterior chamber depth (ACD) were assessed at baseline, mid-intervention, and post-intervention. Significant time effects and time × group interactions were observed for UDVA and KVA (all *p* < 0.05). Although no between-group differences were found at baseline or mid-intervention, the experimental group demonstrated significantly better UDVA and KVA than the control group after the intervention. Significant time effects were observed for AL, CC, and ACD, but no significant between-group differences were detected. These findings suggest that physical exercise combined with accommodative training can effectively enhance visual function in young children, while ocular biometric parameters maintain stable growth trajectories consistent with normal pediatric development.

## 1. Introduction

Myopia has emerged as a pressing global public health concern. According to the World Health Organization, there are approximately 312 million children and adolescents worldwide affected by myopia, and the prevalence is projected to rise sharply if effective preventive and control measures are not implemented [1,2]. In China, the prevalence of myopia among children and adolescents remains high and continues to occur at younger ages. The latest national surveillance data show that in 2022, the overall myopia rate among Chinese children and adolescents reached 51.9%, with rates of 36.7%, 71.4%, and 81.2% in primary, junior, and senior high school students, respectively [3]. The widespread occurrence of myopia not only impairs academic performance and social functioning in youth but also limits long-term developmental potential [4], posing a major challenge to the implementation of the “Healthy China” strategy.

Against this backdrop, visual health has become a crucial component of overall health in children and adolescents. The essence of visual health lies in the stability of multiple indicators that collectively reflect the functional integrity of the visual system. Uncorrected distance visual acuity (UDVA) and kinetic visual acuity (KVA) are core functional measures for evaluating visual performance. UDVA reflects the ability to identify distant targets under static conditions, directly affecting children’s academic efficiency and quality of life [5]. KVA, on the other hand, represents the ability to identify and track moving targets, which depends on ciliary muscle accommodation, ocular motor control, and the efficiency of visual information processing [6], which are also essential for learning and sports participation. Although UDVA and KVA share a moderate positive correlation—with KVA often serving as a predictive indicator for future UDVA trajectories—functional dissociation between them remains common [7,8,9]. Because dynamic tracking is exceptionally sensitive to early ciliary fatigue, KVA performance shifts frequently provide early warning signals before overt static acuity declines manifest [10]. Therefore, combined evaluations of UDVA and KVA provide a comprehensive, complementary profile of visual function performance, serving as sensitive indicators for pediatric visual health interventions.

Visual health, however, is not solely determined by functional indicators; it is also closely related to the development of the refractive system and ocular structures. Axial length (AL) is a key biometric determinant of refractive status and reflects the developmental state of the eyeball [11]. With growth, AL naturally elongates, and the posterior retina experiences increasing mechanical tension. Prolonged near work and excessive screen exposure can accelerate axial elongation, leading to retinal stretching and thinning, degraded image quality [12,13]. Nevertheless, AL alone cannot fully explain refractive changes. During early ocular development, corneal curvature (CC) may flatten as a compensatory mechanism to maintain refractive balance. When axial elongation exceeds a certain threshold, however, corneal compensation fails, resulting in changes in visual acuity. CC, a crucial component of the refractive system, affects refractive power—excessively steep or flat corneas can alter the focal position of light, leading to myopia, hyperopia, or astigmatism. Meanwhile, anterior chamber depth (ACD) reflects geometric and positional changes in the crystalline lens and is closely associated with AL and refractive state. ACD typically deepens with age until ocular growth stabilizes in adolescence, and variations in ACD reflect structural changes during ocular growth. These biometric parameters—AL, CC, and ACD—are in dynamic development during childhood and are influenced by both genetic predisposition and environmental factors. Thus, understanding their developmental trajectories is crucial for exploring visual health issues and evaluating the effects of preventive interventions.

In recent years, physical exercise has been increasingly recognized as an effective approach to promoting visual health and enhancing visual function in children and adolescents. Studies have shown that regular participation in physical and outdoor activities is significantly associated better visual acuity outcomes and may influence both visual function and ocular structures through multiple pathways. First, exercise enhances systemic and ocular blood circulation, improving retinal oxygenation and nutrient delivery. Empirical evidence suggests that engaging in at least 60 min of moderate-to-vigorous physical activity per day can substantially benefit visual function [14]. Second, increased outdoor exposure stimulates retinal dopamine secretion and helps regular ocular growth [11]. Furthermore, the physiological rationale linking accommodative training to enhanced visual function lies in the dynamic regulation of the ciliary muscle [15,16]. Prolonged near work often leads to accommodative lag, transient accommodative spasm, and dysfunction in the coupling between accommodation and vergence, which manifests as degraded image clarity and visual fatigue [17,18,19,20,21,22]. Accommodative training operates on the principle of repeated near-to-far focus shifting, which promotes alternating contraction and relaxation of the ciliary muscle, thereby enhancing accommodative flexibility, accuracy, and endurance [23]. Many interactive physical activities (such as basketball, table tennis, and racquet sports) naturally mirror the biomechanics of formal accommodative training. These sports mandate continuous tracking of dynamic targets and rapid gaze shifts across varying distances, engaging both the accommodative and oculomotor systems under outdoor or well-lit environments [24,25]. It should be noted that in the context of the present intervention, “accommodative training” refer to the functional design of dynamic exercise movement incorporating distance transitions, rather than a confirmed physiological mechanism, as direct accommodative parameters (e.g., amplitude or facility of accommodation) were not measured directly. However, it should be acknowledged that while accommodative exercise effectively relieves ciliary muscle tension and enhances functional responsiveness, current clinical evidence supporting accommodative training as a direct intervention for modifying refractive error or halting structural axial elongation remains limited. Therefore, in the present study, physical exercise combined with accommodative trainingwas conceptualized not as a means to alter baseline refractive status, but as a practical approach to relieve accommodative fatigue and optimize functional visual capacity in early primary school children. Empirical evidence further indicated a significant positive correlation between regular participation in physical activity and visual health among youth. Systematic exercise interventions not only help alleviate transient visual impairment caused by accommodative spasm (pseudomyopia) but also improve ciliary muscle function [26]. However, current findings remain inconsistent, especially regarding the effects of exercise on structural parameters such as AL, CC, and ACD, and the underlying mechanisms of these effects remain to be clarified.

In summary, physical exercise serves as a controllable and practical intervention for visual health promotion among children and adolescents. Most existing studies have focused isolated visual indicators, with limited attention to the combined evolution of functional and structural parameters. Moreover, longitudinal intervention studies targeting lower-grade primary school students are still scarce. Therefore, this study conducted a 32-week randomized longitudinal intervention among first-grade students (aged 6–7). Specifically, our primary hypothesis was that physical exercise combined with accommodative visual tasks would yield significantly greater improvements in visual functional performance (UDVA and KVA) compared to conventional physical exercise alone. Concurrently, we sought to evaluate whether this combined training could exert detectable regulatory effects on ocular biometric parameters (AL, CC, and ACD) alongside baseline physiological growth trajectories in this age group. By clarifying both functional adaptations and structural responses, this study aims to provide empirical evidence for pediatric visual health promotion and offer practical guidance for school-based visual interventions.

## 2. Materials and Methods

### 2.1. Participants

A cluster sampling method was used to recruit four first-grade classes (total *N* = 163) from an elementary experimental school in Suzhou, China. To minimize selection bias, these classes were randomly allocated to either an experimental group (*n* = 82, 2 classes), or a control group (*n* = 81, 2 classes) using a simple draw of lots, which was conducted by an independent researcher not involved in data collection or intervention delivery. The mean age of all participants was 6.91 ± 0.75 years (range: 6 to 9 years), and the sample comprised 91 boys with a mean age of 6.92 ± 0.70 years and 72 girls with a mean age of 6.87 ± 0.71 years. Pearson correlation analyses confirmed that baseline age was not significantly associated with any primary visual or ocular biometric parameter (all *p* > 0.05). Written informed consent for participation and measurement was obtained from all students and their legal guardians prior to the study. Students with congenital disorders, cognitive impairments, or abnormal motor function were excluded. In addition, students who had undergone prior vision-correcting interventions (e.g., refractive surgery, orthokeratology) or who had ocular conditions such as high astigmatism or amblyopia were excluded. All remaining participants underwent baseline assessments of visual acuity and ocular biometric parameters. To achieve complete data collection over the follow-up period, a standardized make-up protocol was implemented: children who were absent on designated testing days due to short-term leave or illness were re-evaluated within the same week using identical protocols and calibrated devices. Furthermore, no students transferred out of the participating classes during the 11-month study period. Consequently, the study achieved complete participant retention (100%, *N* = 163) across all follow-up timepoints. This exceptional stability represents a notable methodological strength of our school-based design, where research procedures were seamlessly embedded into the institution’s existing health-surveillance architecture.

The statistical power of the sample size was evaluated using G*Power software (v3.1.9.2) for a repeated-measures design (2 groups × 3 time points). Based on a standard significance level of *α* = 0.05, an assumed medium effect size (*f* = 0.25), and the total sample size of N = 163 (*n*_Experimental_ = 82, *n*_Control_ = 81), an a priori power analysis indicated a statistical power (1 − *β*) > 0.99 (Numerator *df* = 2, Denominator *df =* 322, Critical *F* = 3.024, noncentrality parameter *λ* = 61.125). This confirms that the study sample size provided sufficient sensitivity to detect significant differences in both visual function and ocular biometric parameters prior to data collection.

### 2.2. Study Design and Interventions

The intervention was designed based on the principle of ciliary muscle accommodation training, aligned with the learning objectives and developmental characteristics of first-grade students defined in the Compulsory Education Physical Education and Health Curriculum Standards (2022 edition). Following the school’s regular teaching schedule, both groups participated in four 40-min physical education classes per week over one academic year (32 weeks). The experimental group received physical exercise combined with accommodative training sessions, integrating near–far visual tasks into standard physical activities, while the control group engaged in regular physical exercise of the same intensity and duration without visual tasks.

The exercise combined with accommodative training program primarily comprised athletics activities (walking, running, jumping, throwing) supplemented by ball sports (basketball, football). The core component was alternated near–far visual tasks designed to train ciliary muscle accommodation. Specifically, during the basic skills and fitness segments of each lesson, participants performed alternating near (0.3 m) and far (3.0 m) visual tracking tasks. One training set consisted of 30 near–far switching trials, with each visual stimulus presented for 3 s [5,27]. Target size was dynamically adjusted according to the students’ average visual acuity (e.g., for mean acuities of 4.9–5.0, far-target sizes of approximately 9.16–7.27 mm were used), whereas the near target size was fixed at 4.59 mm. Targets were presented in two formats: (1) target cards affixed to markers or sports equipment and identified while participants were in motion, and (2) handheld far-target cards presented by a designated group leader who maintained about 3 m distance from the participant to ensure standardization.

Each experimental class was organized into four heterogeneous learning subgroups. A responsible pupil was selected as group leader for each subgroup to manage training organization and to standardize target presentation timing. At the beginning of the intervention, instructors used guided instruction to explain the rationale of accommodative training and to ensure students understanding of task requirements. Throughout the intervention, teachers monitored training fidelity and participant engagement. To address declines in participation observed during mid- to late-intervention, dynamic adherence strategies were implemented, including content adjustments, refinement of target cards, incentive schemes (e.g., small rewards), and periodic changes in training environment to sustain motivation and compliance.

Students in the control classes received the standard physical-education curriculum as scheduled by the school; training content matched that of the experimental classes in terms of skill units and physical activity load but did not include any structured visual tasks. Measurements were taken at three time points: baseline (T1, late February 2024), mid-intervention (T2, late June 2024), and post-intervention (T3, early January 2025). At each time point, UDVA, KVA, AL, CC, and ACD were measured according to standardized procedures. The 32-week intervention was conducted over an 11-month calendar span, comprising two 16-week structured in-school phases across two academic semesters separated by a 7–8 week summer break. Specifically, the first 16-week intervention phase took place during the spring semester. Following the summer break, the second 16-week intervention phase was completed during the fall semester. To ensure continuity and minimize confounding effects of holiday-related near-work, a structured home-based physical activity program (at least 30 min of daily outdoor exercise) was enforced under parent-teacher supervision during the summer break.

Due to the practical nature of school-based physical education interventions, participants and class instructors could not be blinded to group allocation. However, strict masking was enforced for data collection: all outcome assessors conducting the visual acuity tests and ocular biometric measurements were fully masked to participants’ group assignments throughout all testing sessions. Test administrators were provided solely with de-identified participant identification numbers and had no prior knowledge of class or group status. By collecting data across these three designated time points for both intervention and control cohorts, this study established a 2 (Group: Experimental vs. Control) × 3 (Time: Baseline, Mid-intervention, Post-intervention) mixed factorial design to systematically evaluate the main and interaction effects of the physical exercise intervention over time.

### 2.3. Measurements of Visual Acuity and Biometric Parameters

UDVA was measured using a standard logarithmic visual acuity light box (GB11533, National Standard of China). The testing environment was kept quiet, clean, and free from distractions. Participants stood 5 m from the chart, and visual acuity was tested for the right and left eyes separately. During testing, the examiner pointed to the “E” optotypes on the chart, and the participant—while covering the non-tested eye with an occluder—indicated the direction of the optotype gap using their hand. Participants continued reading progressively smaller lines until more than half of the optotypes in a line were misidentified. Subjects who wore glasses were asked to remove them prior to testing. Throughout the test, participants were instructed not to squint or press the covered eye. The poorer visual acuity between the two eyes was recorded as the participant’s final UDVA score.

KVA was assessed using the XP.14-TD-J905 Kinetic Visual Acuity Tester (manufactured by Shanghai Tuofeng Automation Technology Co., Ltd., Shanghai, China). During the test, participants sat in front of the device with both eyes aligned to the viewing aperture. Upon initiation by the examiner, a “C”-shaped optotype appeared within the aperture and simulated an approach from a distance of 50 m toward the observer at an approximate velocity of 30 km/h. The optotype’s opening direction (up, down, left, or right) varied randomly. Participants were instructed to move the joystick immediately in the perceived direction of the gap. Each participant completed three trials, with a 30-s rest between tests. The mean of the three measurements was used as the final KVA score.

AL, CC, and ACD were measured using an optical biometer (IOL Master 500, Carl Zeiss Meditec AG, Jena, Germany). Participants stood naturally in front of the instrument, with the chin placed on the chin rest and the forehead against the headrest. The examiner adjusted the height of the device so that the participant’s eye was aligned with the viewing aperture. Measurements were taken first for the left eye and then for the right eye. Participants were instructed to keep their eyes open, avoid blinking, and fixate on the red fixation light within the viewing field. Once optimal focus was achieved, the instrument automatically recorded the AL, CC, and ACD values. Measurement results were printed immediately after completion. For statistical analysis, data from the eye corresponding to the poorer UDVA were used to represent each participant’s ocular measurements.

Due to the high inter-eye correlation within individuals, analyzing both eyes as independent data points introduces significant statistical dependency. Therefore, consistent with established protocols in pediatric myopia and vision intervention research, the “poorer eye” was selected as the representative eye for each participant in all statistical analyses. This approach targets the clinically most vulnerable eye while avoiding potential ceiling effects associated with the better eye.

### 2.4. Data Processing and Statistical Analysis

All statistical analyses were performed using SPSS 26.0. Data were expressed as mean ± standard deviation (M ± SD). Preliminary baseline homogeneity between the experimental and control groups was evaluated using independent-samples *t*-tests for continuous variables and Pearson’s chi-square (χ^2^) tests for categorical variables. Baseline continuous variables satisfied the assumptions of normality via the Shapiro–Wilk test (UDVA: *W* = 0.996, *p* = 0.979; KVA: *W* = 0.990, *p* = 0.794; AL: *W* = 0.979, *p* = 0.187; CC: *W* = 0.974, *p* = 0.099; ACD: *W* = 0.996, *p* = 0.964) and homoscedasticity via Levene’s test based on mean (UDVA: *F* = 2.172, *p* = 0.142; KVA: *F* = 0.022, *p* = 0.883; AL: *F* = 2.134, *p* = 0.146; CC: *F* = 2.713, *p* = 0.101; ACD: *F* = 0.001, *p* = 0.971), and sex distribution showed no significant baseline difference between groups (χ^2^ = 0.060, *p* = 0.806). Furthermore, preliminary factorial analyses incorporating Sex as a potential moderator revealed no significant main effect of Sex nor any Sex × Group interaction across primary outcomes (all *p* > 0.05); thus, Sex was not retained as a covariate in the primary outcome models.

A 2 (Group: experimental, control) × 3 (Time: pre-, mid-, and post-test) mixed-design repeated-measures ANOVA was applied to analyze the main effects of group and time, as well as their interaction effects, across five primary dependent variables (UDVA, KVA, AL, CC, and ACD). Each dependent variable was evaluated as an independent outcome family based on its distinct functional or structural properties. To stringently control the family-wise Type I error rate, Bonferroni corrections were applied to all post hoc pairwise comparisons and simple-effects analyses following significant interaction effects.

Assumptions for repeated-measures ANOVA were formally evaluated prior to hypothesis testing. Normal distribution of residuals was re-confirmed via Shapiro–Wilk testing and visual inspection of Q-Q plots. Mauchly’s Test of Sphericity was conducted to assess the sphericity assumption; in cases where sphericity was violated (*p* < 0.05), the Greenhouse–Geisser correction was applied to adjust degrees of freedom and corresponding *p*-values.

To evaluate the potential impact of cluster allocation (class-level clustering) on primary statistical inferences, a sensitivity analysis was performed using Linear Mixed-Effects Models (LMM). In these models, Class was specified as a random intercept to model class-level variability, while Group, Time, and the Group × Time interaction were treated as fixed effects. Model parameters were estimated using restricted maximum likelihood.

### 2.5. Ethics

The study was conducted in accordance with the tenets of the Declaration of Helsinki and was approved by the Ethics Committee of Soochow University (No. SUDA20201010H01). The studies were conducted in accordance with the local legislation and institutional requirements. Written informed consent for participation in this study was provided by the participants’ legal guardians/next of kin.

## 3. Results

### 3.1. Baseline Characteristics of Participants

Independent-samples *t*-tests were conducted to analyze the baseline data of students in the experimental and control groups (Table 1). The results indicated no significant differences in visual acuity (UDVA and KVA) and ocular biometric parameters (AL, CC, and ACD) between the two groups prior to the intervention (*p* > 0.05).

### 3.2. Effects of Physical Exercise on Students’ Visual Acuity

#### 3.2.1. Effects of Physical Exercise on Student’s UDVA

A two-way mixed-design repeated-measures ANOVA was conducted to examine the effects of the intervention on UDVA across three testing sessions. As shown in Table 2, repeated-measures ANOVA revealed a significant main effect of time (*F* (2,322) = 8.392, *p* < 0.001, partial η^2^ = 0.050), indicating that overall UDVA changed significantly across the 32-week period regardless of group assignment. The main effect of group was not significant (*F* (1,161) = 0.929, *p* > 0.05, partial η^2^ = 0.006). Crucially, a significant time × group interaction was observed (*F* (2,322) = 4.042, *p* < 0.05, partial η^2^ = 0.024), demonstrating that the longitudinal trajectory of UDVA differed significantly between the two groups over time. To further specify the nature and direction of these group-specific longitudinal changes, simple effect analyses and post hoc pairwise comparisons were subsequently conducted.

A simple-effects analysis was further conducted by fixing either the group (experimental/control) or time (T1, T2, T3) variables to examine their differential influence on UDVA. For the between-group comparisons (Table 3), no significant difference was observed between the experimental and control groups at T1 (*P*_adj_ = 0.485) or T2 (*P*_adj_ = 0.452). Following Bonferroni adjustment (*k* = 3), the between-group difference at T3 exhibited a marginally significant trend favoring the experimental group (*P*_adj_ = 0.069). Crucially, for the within-group longitudinal comparisons, time had a highly significant main effect on UDVA in the experimental group (*p* < 0.001), whereas no significant longitudinal change occurred in the control group (*p* = 0.574). This contrast confirms that the overall intervention efficacy was primarily driven by continuous longitudinal gains within the experimental group rather than natural shifts in the control cohort.

Post hoc pairwise comparisons with Bonferroni correction further detailed the temporal pattern of this improvement in the experimental group: UDVA improved significantly from T1 to T2 (*P*_adj_ = 0.007) and from T1 to T3 (*P*_adj_ = 0.002). However, the change from T2 to T3 showed a diminishing return trend and did not reach statistical significance after adjustment (*P*_adj_ = 0.203).

#### 3.2.2. Effects of Physical Exercise on Student’s KVA

Similarly, as shown in Table 4, the mixed-design repeated-measures ANOVA for KVA revealed a significant main effect of time (*F* (2,322) = 12.439, *p* < 0.001, partial η^2^ = 0.067), indicating overall temporal variation across the 32-week period. The main effect of group was also significant (*F* (1,161) = 4.902, *p* < 0.05, partial η^2^ = 0.030). Crucially, a significant time × group interaction was observed (*F* (2,322) = 5.299, *p* < 0.01, partial η^2^ = 0.062), demonstrating that the longitudinal trajectory of KVA differed significantly between the two groups over time. To further specify the nature and direction of these group-specific shifts, simple effect analyses and post hoc pairwise comparisons were subsequently conducted.

For the between-group comparisons (Table 5), no significant difference was observed between the experimental and control groups at baseline T1 (*P*_adj_ = 0.672) or mid-test T2 (*P*_adj_ = 0.206). However, a highly significant difference emerged at post-test T3 (*P*_adj_ < 0.001), with the experimental group markedly outperforming the control group. For the within-group longitudinal comparisons, time had a highly significant main effect on KVA in the experimental group (*p* < 0.001), whereas no significant change occurred in the control group (*p* = 0.472). This contrast directly confirms that the intervention yielded a substantial net improvement in kinetic visual acuity.

Subsequent pairwise comparisons with Bonferroni correction revealed that, within the experimental group, KVA showed a progressive trend toward improvement from T1 to T2 (*P*_adj_ = 0.086) and achieved highly significant overall gains from T1 to T3 (*P*_adj_ < 0.001). Furthermore, KVA continued to improve significantly during the second half of the intervention from T2 to T3 (*P*_adj_ = 0.032), demonstrating sustained functional visual enhancement across the entire intervention period.

### 3.3. Effects of Physical Exercise on Students’ Ocular Biometric Parameters

#### 3.3.1. Effects of Physical Exercise on Student’s AL

A two-way mixed-design repeated-measures ANOVA was conducted to analyze students’ AL across testing stages. As shown in Table 6, the main effect of time was highly significant (*F* (2,322) = 23.474, *p* < 0.001, partial η^2^ = 0.127), reflecting significant, time-dependent longitudinal changes in AL over the 32-week period across all participants regardless of group allocation. Neither the main effect of group (*F* (1,161) = 0.052, *p* > 0.05, partial η^2^ = 0.001) nor the time × group interaction (*F* (2,322) = 0.086, *p* > 0.05, partial η^2^ = 0.001) was statistically significant.

The absence of a significant interaction confirms that the longitudinal trajectory of AL elongation did not differ between the intervention and control cohorts, indicating that the combined exercise did not exert an additive moderating effect on axial growth over 32 weeks. Consequently, analysis was confined to the main effect of time across the pooled sample. Post hoc pairwise comparisons with Bonferroni correction demonstrated progressive axial elongation: AL at T2 (*P*_adj_ = 0.011) and T3 (*P*_adj_ < 0.001) was significantly greater than at baseline T1, with T3 also significantly exceeding T2 (*P*_adj_ < 0.001). This uniform increase reflects normative, age-related biological ocular growth characteristic of 6-to-7-year-old children rather than an intervention-driven effect.

#### 3.3.2. Effects of Physical Exercise on Student’s CC

A two-way mixed-design repeated-measures ANOVA was conducted to analyze the CC data of students across different testing stages. As shown in Table 7, the main effect of time was significant (*F* (2,322) = 4.132, *p* < 0.05, partial η^2^ = 0.025), indicating that all participants exhibited significant, time-dependent longitudinal changes in CC over the 32-week period. The main effect of group was not significant (*F* (1,161) = 0.082, *p* > 0.05, partial η^2^ = 0.001), and the time × group interaction was also not significant (*F* (2,322) = 0.001, *p* > 0.05, partial η^2^ = 0.000).

Because the interaction was non-significant, simple-effects analyses were omitted, confirming that corneal biomechanics followed identical trajectories in both groups. Main-effect post hoc pairwise comparisons (Bonferroni-adjusted) revealed that CC values remained highly stable between short-term intervals—showing no significant difference between T1 and T2 (*P*_adj_ = 0.375) or between T2 and T3 (*P*_adj_ = 0.610). Nevertheless, across the overall 32-week span, CC at T3 was significantly lower (flatter) than at baseline T1 (*P*_adj_ = 0.012) in the combined cohort. This slight overall flattening illustrates the normative physiological corneal compensation mechanism that typically accompanies developmental axial growth in primary school children.

#### 3.3.3. Effects of Physical Exercise on Student’s ACD

A two-way mixed-design repeated-measures ANOVA was performed to analyze changes in ACD across different testing stages in both groups. As presented in Table 8, the main effect of time was significant (*F* (2,322) = 3.466, *p* < 0.05, partial η^2^ = 0.019), indicating statistically significant longitudinal changes in ACD over the 32-week period. Neither the main effect of group (*F* (1,161) = 0.005, *p* > 0.05, partial η^2^ = 0.000), nor the time × group interaction (*F* (2,322) = 0.112, *p* > 0.05, partial η^2^ = 0.001) reached statistical significance.

The non-significant interaction confirms that physical exercise combined with accommodative visual tasks did not alter structural anterior chamber dimensions relative to standard physical activity. Post hoc pairwise comparisons revealed that ACD at T2 showed no significant difference from T1 (*P*_adj_ = 0.844) or T3 (*P*_adj_ = 0.379). However, ACD at T3 was significantly greater than at baseline T1 (*P*_adj_ = 0.046) across the pooled sample.

### 3.4. Sensitivity Analysis for Class-Level Clustering

To evaluate the impact of class-level clustering on our primary outcomes, a linear mixed-effects model (LMM) with class specified as a random intercept was performed as a sensitivity analysis. A total of 4 classes (2 in the intervention arm and 2 in the control arm) were modeled. Model convergence was successfully achieved under Restricted Maximum Likelihood (REML) estimation, verified by standard convergence criteria with parameter changes tolerance below 10^−6^. Main effects for Group and Time were consistent with primary models, and the key parameter of interest—the Group × Time interaction—remained robust across all outcomes after adjusting for class-level clustering (Table 9).

As detailed in Table 9, the random intercept variance for classes was minimal across all parameters. After accounting for class-level clustering, the Group × Time interaction effects remained statistically significant for functional vision parameters, including UDVA (*F* = 2.791, *p* = 0.041) and KVA (*F* = 3.968, *p* = 0.020). For ocular biometric parameters (AL, CC, and ACD), class-level random intercept variances were estimated at 0.000000 (SE = 0.000000), with non-significant interaction effects (*p* > 0.05). However, given that only four intact clusters were included, the LMM algorithm has limited statistical power to isolate between-class variance from residual error, often resulting in REML boundary solutions pinned at zero. Therefore, these zero-variance estimates should be interpreted as inconclusive regarding the true presence of clustering effects rather than definitive confirmation of zero between-class variability. Nonetheless, the overall pattern of results remains highly consistent across fixed-effect specifications.

## 4. Discussion

### 4.1. Comparative Analysis of Changes in Students’ Visual Acuity Before and After the Exercise Intervention

This study demonstrated that after 32 weeks of physical exercise combined with accommodative training, students in the experimental group showed significant improvements in both UDVA and KVA at T2 and T3 compared with T1, with further gains observed from T2 to T3. In contrast, although students in the control group exhibited slight increases in UDVA and KVA across testing stages, these changes were not statistically significant. Both groups experienced modest overall improvements, indicating that regular physical exercise itself exerts a beneficial influence on children’s visual health. Regular exercise has been shown to enhance cardiovascular function and ocular blood circulation, improve oxygenation and metabolism of the ciliary body and retina, and reduce prolonged near-work demands, thereby alleviating ciliary muscle tension and visual fatigue, ultimately supporting the maintenance of visual function in children and adolescents [28,29]. Thus, the fundamental role of physical activity in maintaining and enhancing visual health among primary school students should not be overlooked.

However, the more pronounced improvement observed in the experimental group suggest that incorporating reinforced visual tasks into conventional physical exercise yields a stronger intervention effect. This enhancement likely arises from the multi-level activation and integrative engagement of the visual system during visually demanding exercise. UDVA primarily reflects the ability to identify distant objects under static conditions. Previous studies have shown that repeated stimulation of near-to-far accommodative shifts (e.g., dynamic visual tracking training) can not only effectively improve KVA but also enhance the endurance and sensitivity of ciliary muscle accommodation, thereby indirectly contributing to improvements in UDVA [27]. Moreover, KVA has been identified as a mediating variable that plays a crucial bridging role in the relationship between physical activity and visual health among children and adolescents [7]. Therefore, physical exercise combined with accommodative training plays an important role in promoting ocular functional development and maintaining visual health. Although direct accommodative parameters were not measured in this study, the frequent gaze shifts and dynamic target tracking inherent in the intervention are movement patterns designed to stimulate ciliary muscle function, which may explain the stronger observed effects on both UDVA and KVA.

In addition, exercise combined with accommodative training appears to influence vision through deeper neurocognitive mechanisms [30]. Tasks involving visual tracking and saccadic recognition increase oculomotor activity and improve smooth pursuit and saccadic accuracy, thereby strengthening dynamic target detection and directly promoting KVA performance [31]. Empirical evidence indicated that repeated engagement in visual training can induce neuroplastic changes within the neural circuits responsible for visual processing, thereby enhancing detection, discrimination, and response selection, ultimately enabling faster and more accurate performance in visual tasks [32]. Meanwhile, regular exercise upregulates the expression of brain-derived neurotrophic factor (BDNF) and strengthens neural connectivity, enhancing attentional resources and information processing efficiency, which allow children to exhibit superior perceptual–decision responses during visual tasks [33]. At the ocular tissue level, this systemic integration converges onto a unified retina–choroid–sclera signaling cascade that serves as the downstream physiological foundation supporting these functional vision gains. Physical activity combined with accommodative tasks provides dual-stream stimulation: dynamic target tracking optimizes ciliary kinetics to reduce hyperopic retinal defocus [34], while physical exertion directly boosts ocular perfusion and stimulates retinal dopamine secretion [35]. These optical–mechanical and neurochemical pathways converge at the choroid-scleral interface to enhance choroidal blood flow and alleviate scleral hypoxia [36], which directly prevents scleral extracellular matrix (ECM) degradation and maintains tissue integrity [37]. Crucially, this oxygenated, metabolic-optimized tissue microenvironment protects retinal neural processing and prevents ciliary fatigue, thereby providing the essential structural and physiological substrate that sustains improvements in UDVA and drives continuous gains in KVA.

A further comparison of changes in UDVA and KVA between groups revealed no significant differences between the experimental and control groups at T1 and T2. Following Bonferroni adjustment, the experimental group demonstrated a highly significant advantage in KVA at T3, alongside a modest trend toward higher UDVA. Within the experimental group, visual acuity improvements accrued rapidly from T1 to T2. Thereafter, UDVA gains plateaued from T2 to T3, reflecting a typical physiological stabilization phase following initial neuromuscular and visual adaptations. In contrast, KVA demonstrated sustained and significant continuous improvements throughout the entire intervention period, maintaining robust gains from T2 through T3. By T3, the cumulative impact of the intervention became evident, producing systematic and measurable improvements in the experimental group that were not observed in the control group. This result further confirms the long-term efficacy and practical effectiveness of physical exercise combined with accommodative training in improving students’ UDVA and KVA. However, when interpreting these functional visual gains, it is essential to distinguish clinical significance from pure statistical significance by considering the practical magnitude of the effect sizes. The Group × Time interaction for KVA exhibited a moderate effect size, whereas UDVA demonstrated a relatively small effect size. In real-world school-based lifestyle interventions, modest effect sizes are typical given the multifactorial influences on pediatric visual development. From a clinical and public health perspective, even a small, incremental maintenance or improvement in visual acuity provides meaningful clinical protection by buffering primary school children against the normative, progressive age-related decline in vision associated with intense academic near-work. These metric-specific trajectories carry concrete practical implications for refining pediatric visual health guidelines. The rapid initial improvement in UDVA provides immediate positive reinforcement to boost intervention compliance, while the subsequent plateau indicates a stable maintenance phase rather than intervention fatigue, advising health practitioners against unnecessarily escalating exercise intensity once static acuity stabilizes. Conversely, the delayed and continuous gains in KVA demonstrate that dynamic visuomotor tracking requires sustained engagement to consolidate ciliary accommodation. Consequently, updated public health guidelines should mandate a minimum effective intervention threshold, emphasizing to educators and parents that dynamic visual protection requires a cumulative, multi-term commitment.

### 4.2. Comparative Analysis of Changes in Students’ Ocular Biometric Parameters Before and After the Exercise Intervention

The results of this study showed that 32 weeks of physical exercise produced a significant main effect of time on students’ AL, CC, and ACD, but no significant group effect or group-by-time interaction. These results indicate that the measured structural changes primarily reflect natural physiological development across the observation period, and that the addition of accommodative training to physical exercise did not produce measurable differential effects on these anatomical indices compared to regular physical exercise. Concretely, AL increased progressively, CC showed a gradual flattening, and ACD deepened over time—patterns consistent with normal ocular growth in childhood.

AL is a principal biometric determinant of refractive development. The progressive increase in AL with age is a normal physiological process: the mean AL in elementary school students is about 23.9 mm, whereas in university students it can reach 25.8 mm [38], underscoring the close coupling between ocular growth and refractive development. Previous observational studies have suggested that outdoor activity may help moderate axial growth in children. However, in the present study, no statistically significant between-group differences were detected in AL elongation rates. This suggests that within a 32-week period, the combined intervention did not exert a detectable additive effect on suppressing axial growth beyond that of regular physical activity. While physical activity enhances systemic and ocular blood circulation and reduces visual fatigue [33], macro-structural parameters like AL in 6-to-7-year-old children appear to be strongly dictated by baseline physiological growth trajectories.

The results also revealed a gradual decline in CC over time, reflecting an increase in the corneal curvature radius and a relative flattening of the cornea. This finding aligns with prior research. Lu found that CC decreased progressively with grade level, with first-grade students exhibiting the highest CC and sixth-grade students the lowest [39]. Similarly, a cross-sectional study involving 12,025 primary school students reported that AL increased progressively with grade level, whereas CC remained relatively stable [40]. During ocular development, an optical compensation mechanism may operate: a decrease in CC partially offsets axial elongation, helping maintain retinal focus [41]. Genetic studies further confirm a developmental linkage between AL and CC, showing that a 1 mm increase in CC is accompanied by an average increase of approximately 2.9 mm in AL, thereby partially compensating for refractive imbalances [42]. Previous intervention studies using table tennis and badminton among myopic children and adolescents also found no substantial changes in CC [43]. Therefore, the lack of group differences in CC confirms that neither conventional exercise nor exercise combined with accommodative training alters baseline corneal curvature, reinforcing the view that corneal biomechanics are resistant to short-term behavioral interventions.

Regarding ACD, a gradual deepening was observed across testing timepoints, which corresponds to natural anterior segment maturation, including lens thinning and anterior chamber widening during child growth [40]. The average annual increase in ACD is approximately 0.02–0.03 mm, which correlates with age, height, and refractive status. During accommodation, anterior ciliary contraction causes lens thickening and curvature increase, resulting in a shallower anterior chamber [44]. The physical exercise combined with accommodative training in this study involved frequent near–far visual alternations, although such accommodative training involves dynamic target tracking and ciliary muscle contraction that transiently affect lens positioning, these functional tasks did not lead to permanent structural modifications in anterior chamber dimensions, resulting in no significant group differences.

It is noteworthy that no significant differences were found between the exercise combined with accommodative training and conventional exercise groups in AL, CC, and ACD. This finding may be attributed to two main factors. First, the participants—children aged six to seven years—were in a period of rapid ocular development primarily governed by genetic and natural growth factors, during which non-invasive behavioral interventions modulate function rather than induce drastic morphological divergence between cohorts. Second, while the exercise combined with accommodative training effectively enhanced ciliary muscle responsiveness, such non-pharmacological behavioral modalities operate primarily through physiological regulation—such as relieving accommodation fatigue and promoting ocular microcirculation—rather than direct physical alteration or remodeling of ocular tissue structures. Consequently, functional gains occur rapidly, whereas biometric parameters maintain a stable, regulated growth trajectory parallel across groups.

In summary, while the 32-week intervention did not produce significant between-group differences in AL, CC, or ACD, the significant functional gains in UDVA and KVA highlight the practical value of integrating accommodative training into physical exercise. Given that first-grade students aged six to seven are in a phase of rapid ocular growth dominated by hereditary and developmental factors, short-term exercise interventions are unlikely to produce immediate structural changes. Nonetheless, these findings do not imply that physical exercise combined with accommodative training is ineffective. Long-term, regular engagement in visually integrated physical activity may gradually produce stable structural regulation effects during later developmental stages (ages 8–10). From a practical screening perspective, because macro-structural biometric parameters follow steady, normative biological growth trajectories, routine school-based vision screening protocols do not require high-frequency structural biometric testing. Practical guidelines should instead recommend extended annual monitoring intervals for structural parameters like axial length, while focusing routine, mid-term follow-ups on functional visual tracking via UDVA and KVA to optimize public health resource allocation. Future research should therefore extend intervention duration and incorporate additional indicators—such as choroidal thickness, aqueous humor dynamics, and corneal biomechanics—to elucidate the comprehensive regulatory mechanisms of physical exercise on ocular structural development.

### 4.3. Shortcomings and Prospects

Although this study provides preliminary evidence regarding the effects of physical exercise on visual acuity and ocular biometric parameters among primary school students, several limitations should be acknowledged. (1) Due to constraints related to the duration of the intervention, organizational feasibility, and management costs, this study recruited only four first-grade classes from one elementary school in Suzhou, resulting in a relatively limited sample size. This may have reduced the statistical power and external generalizability of the findings. (2) Cycloplegic refraction was not performed, and key clinical variables such as objective refractive error and accommodative amplitude were not measured due to logistical and instrumentation limitations. Because cycloplegia is required for definitive myopia diagnosis in young children, the absence of refractive status evaluation limits a comprehensive understanding of the precise optical mechanisms and prevents direct conclusions regarding myopia control. (3) The intervention period lasted only 32 weeks, which may have been insufficient to induce substantial structural changes in ocular biometry or to capture the long-term effects of exercise on myopia prevention and visual development. (4) The intervention lacked objective real-time monitoring of individual accommodative responses, fixation accuracy, and eye-movement kinetics during physical activity. Although near–far visual tasks were structured within classes, individual variations in actual accommodative demands could not be continuously quantified via wearable ocular tracking sensors. (5) Crucially, the outcome battery lacked validated clinical parameters of ocular accommodative function. Although our intervention was conceptualized as “accommodative training” targeting ciliary-muscle dynamics, none of the primary outcomes (UDVA, KVA, AL, CC, ACD) constitutes an established clinical diagnostic test of accommodative function per se. As highlighted in the systematic review by Cacho-Martínez et al. [45], the primary clinical signs with robust diagnostic validity for accommodative dysfunctions are the amplitude of accommodation (AMP) and monocular accommodative facility (MAF) for accommodative insufficiency, as well as positive relative accommodation (PRA) for accommodative excess. Without these validated physiological metrics, we cannot definitively rule out alternative, non-accommodative pathways contributing to the observed functional gains in UDVA and KVA, such as enhanced central visual attention, improved oculomotor tracking, or task-learning effects.

Future research should therefore seek to address these limitations from several perspectives First, multi-center, large-scale longitudinal studies across broader age groups and regional backgrounds are needed to enhance the representativeness and external validity of the findings. Second, subsequent investigations should incorporate cycloplegic autorefraction alongside detailed measurements of refractive status to elucidate the precise optical mechanisms underlying visual adaptation and myopia control. Third, extending the intervention and follow-up duration is essential to evaluate the sustained and cumulative effects of exercise on ocular biometric development. Future studies should also explore how different types, frequencies, and intensities of visually integrated physical activity produce distinct outcomes in maintaining and promoting pediatric visual health. Fourth, while the school-based intervention demonstrated high ecological validity and feasibility, objective real-time monitoring of individual accommodative responses, fixation accuracy, and eye-movement kinetics (e.g., via wearable eye-trackers or dynamic photorefractors) was not performed during physical activities. Future multi-center studies should incorporate wearable objective ocular sensors to quantify individual accommodative demands and dose–response relationships. Fifth, direct clinical parameters of ocular accommodation (such as accommodative amplitude, accommodative facility, accommodative lag, and vergence kinetics) were not directly measured in this field trial. Consequently, while functional enhancements in dynamic visual acuity were robustly demonstrated, the underlying neuromuscular and accommodative mechanisms remain inferential. Subsequent studies must incorporate validated, epidemiologically supported clinical tests of accommodative function—specifically measuring the amplitude of accommodation (AMP), monocular and binocular accommodative facility (MAF/BAF), and positive/negative relative accommodation (PRA/NRA)—to directly quantify ciliary-muscle adaptation, verify neuromuscular pathways, and substantiate the proposed physiological mechanisms underlying visually integrated physical activities.

## 5. Conclusions

Regular physical activity that combines with accommodative training demonstrates clear benefits for enhancing children’s visual function, particularly in improving students’ UDVA and KVA. However, no significant effects were observed on AL, CC, or ACD. These findings suggest that functional adaptations occur rapidly, whereas structural biometric parameters maintain stable, regulated growth trajectories consistent with normal ocular development. Further long-term and large-scale studies incorporating objective refractive assessments are warranted to elucidate the precise pathways and consolidate the practical role of visually integrated exercise in safeguarding children’s visual health.

## Figures and Tables

**Table 1:** Baseline characteristics of participants before the intervention.

Variable		Experimental Group	Control Group	*p* Value
n (students)	Male	45	46	
	Female	37	35	
	Total	82	81	
UDVA		4.903 ± 0.180	4.912 ± 0.149	0.485
KVA		0.315 ± 0.196	0.328 ± 0.200	0.672
AL/mm		22.908 ± 0.519	22.871 ± 0.685	0.700
CC/mm		43.383 ± 0.540	43.405 ± 0.611	0.811
ACD/mm		3.396 ± 0.238	3.406 ± 0.240	0.852

Note: Pearson’s chi-square test revealed no significant difference in sex distribution between groups (χ^2^ = 0.060, df = 1, *p* = 0.806).

**Table 2:** Descriptive statistics and mixed-design ANOVA results for UDVA (M ± SD).

Time	Experimental Group (M ± SD)	Control Group (M ± SD)	Time Effect *F *(*p*)	Group Effect *F *(*p*)	Interaction *F *(*p*)
T1	4.903 ± 0.180	4.912 ± 0.149	8.392 (0.000 ***)	0.929 (0.337)	4.042 (0.037 *)
T2	4.939 ± 0.183	4.921 ± 0.187			
T3	4.997 ± 0.177	4.932 ± 0.237			

Note: partial η^2^ for time effect = 0.050, for group effect = 0.006, and for interaction = 0.024; * indicates *p* < 0.05, *** indicates *p* < 0.001.

**Table 3:** Simple effects analysis of the UDVA interaction effect.

Analysis Type	Time/Group	*F* Value	*p* Value
Between-Group Comparison	T1	0.490	0.485
	T2	0.569	0.452
	T3	3.348	0.069
Within-Group Comparison	Experimental Group	12.747	0.000 ***
	Control Group	0.557	0.574

Note: *P*_adj_ values for simple-effect comparisons between groups were adjusted using the Bonferroni method (*k* = 3). *** indicates *p* < 0.001.

**Table 4:** Descriptive statistics and mixed-design ANOVA results for KVA (M ± SD).

Time	Experimental Group (M ± SD)	Control Group (M ± SD)	Time Effect *F *(*p*)	Group Effect *F *(*p*)	Interaction *F *(*p*)
T1	0.315 ± 0.196	0.328 ± 0.200	12.439 (0.000 ***)	4.902 (0.025 *)	5.299 (0.006 **)
T2	0.393 ± 0.166	0.349 ± 0.269			
T3	0.476 ± 0.191	0.363 ± 0.192			

Note: partial η^2^ for time effect = 0.067, for group effect = 0.030, and for interaction = 0.062; * indicates *p* < 0.05, ** indicates *p* < 0.01, *** indicates *p* < 0.001.

**Table 5:** Simple effects analysis of the KVA interaction effect.

Analysis Type	Time/Group	*F* Value	*p* Value
Between-Group Comparison	T1	0.180	0.672
	T2	1.609	0.206
	T3	14.254	0.000 ***
Within-Group Comparison	Experimental Group	17.083	0.000 ***
	Control Group	0.755	0.472

Note: *P*_adj_ values for simple-effect comparisons between groups were adjusted using the Bonferroni method (*k* = 3). *** indicates *p* < 0.001.

**Table 6:** Descriptive statistics and mixed-design ANOVA results for AL (M ± SD).

Time	Experimental Group (M ± SD)	Control Group (M ± SD)	Time Effect *F *(*p*)	Group Effect *F *(*p*)	Interaction *F *(*p*)
T1	22.908 ± 0.519	22.871 ± 0.685	23.474 (0.000 ***)	0.052 (0.832)	0.086 (0.821)
T2	23.056 ± 0.554	23.041 ± 0.626			
T3	23.226 ± 0.589	23.230 ± 0.431			

Note: partial η^2^ for time effect = 0.127, for group effect = 0.001, and for interaction = 0.001; *** indicates *p* < 0.001.

**Table 7:** Descriptive statistics and mixed-design ANOVA results for CC (M ± SD).

Time	Experimental Group (M ± SD)	Control Group (M ± SD)	Time Effect *F *(*p*)	Group Effect *F* (*p*)	Interaction *F* (*p*)
T1	43.383 ± 0.540	43.405 ± 0.611	4.132 (0.017 *)	0.082 (0.921)	0.001 (0.978)
T2	43.301 ± 0.762	43.296 ± 0.851			
T3	43.243 ± 0.763	43.219 ± 0.768			

Note: partial η^2^ for time effect = 0.025, for group effect = 0.001, and for interaction = 0.000; * indicates *p* < 0.05.

**Table 8:** Descriptive statistics and mixed-design ANOVA results for ACD (M ± SD).

Time	Experimental Group (M ± SD)	Control Group (M ± SD)	Time Effect *F* (*p*)	Group Effect *F* (*p*)	Interaction *F* (*p*)
T1	3.396 ± 0.238	3.406 ± 0.240	3.466 (0.047 *)	0.005 (0.946)	0.112 (0.894)
T2	3.434 ± 0.220	3.423 ± 0.236			
T3	3.457 ± 0.172	3.454 ± 0.207			

Note: partial η^2^ for time effect = 0.019, for group effect = 0.000, and for interaction = 0.001; * indicates *p* < 0.05.

**Table 9:** Comparison of Group × Time Interaction Effects Between Primary Analysis (ANOVA) and Sensitivity Analysis (LMM).

Outcome	Primary Analysis (ANOVA) *F *(*p*)	Sensitivity Analysis (LMM) *F* (*p*)	Class Random Intercept Variance σ^2^ (SE)
UDVA	4.042 (0.037 *)	2.791 (0.041 *)	0.001771 (0.002055)
KVA	5.299 (0.006 **)	3.968 (0.020 *)	0.001478 (0.001818)
AL	0.086 (0.821)	0.052 (0.950)	0.000000 (0.000000)
CC	0.001 (0.978)	0.041 (0.959)	0.000000 (0.000000)
ACD	0.112 (0.894)	0.068 (0.934)	0.000000 (0.000000)

Note: Values presented for ANOVA and LMM represent the Group × Time interaction term. * indicates *p* < 0.05, ** indicates *p* < 0.01.

## Data Availability

The data that support the findings of this study are available on request from the first author. The data are not publicly available because they contain information that could compromise the privacy of research participants. Requests to access the datasets should be directed to R.Y. (yrb@suda.edu.cn).

## Abbreviations

The following abbreviations are used in this manuscript:

UDVA	Uncorrected Distance Visual Acuity
KVA	Kinetic Visual Acuity
AL	Axial Length
CC	Corneal Curvature
ACD	Anterior Chamber Depth
